# Anisotropy Characterization of Metallic Lens Structures

**DOI:** 10.3390/mi12091114

**Published:** 2021-09-16

**Authors:** Yosef T. Aladadi, Majeed A. S. Alkanhal

**Affiliations:** Department of Electrical Engineering, King Saud University, Riyadh 11421, Saudi Arabia; majeed@ksu.edu.sa

**Keywords:** metallic lens, anisotropy, tensorial parameters, impedance matching, losses

## Abstract

This paper presents a full electromagnetic (EM) characterization of metallic lenses. The method is based on the utilization of free-space transmission and reflection coefficients to accurately obtain lenses’ tensorial EM parameters. The applied method reveals a clear anisotropic behavior with a full tensorial directional permittivity and permeability and noticeably dispersive permeability and wave impedance. This method yields accurate values for the effective refractive index, wave impedance, permittivity, and permeability, unlike those obtained by simple methods such as the eigenmode method. These correct cell parameters affect their lens performance, as manifested in a clear level of anisotropy, impedance matching, and losses. The effect of anisotropy caused by oblique incidence on the performance and operation of lens designs is illustrated in a lens design case.

## 1. Introduction

Artificial gradient refractive index (GRIN) materials [1], electromagnetic band-gap (EBG) structures [2], photonic crystals [3], and left-handed materials [4] are broadly classified as metamaterials, which are typically produced from periodic metallic and dielectric structures [5,6]. Among various metamaterials, metallic GRIN metamaterials have properties that are difficult or impossible to achieve with traditional material fabrication methods [7]. Owing to such features, GRIN metamaterials play important roles in designing lenses [6,8,9] and similar devices. They can be utilized as lenses for beam collimation and deflection in the microwave range [10] and as cloaks in the optical range [11]. Metallic parallel-plate waveguide (PPWG) structures are the simplest periodic artificial sub-wavelength structures of GRIN metamaterials to have been adopted for designing lenses in the microwave and sub-terahertz ranges [12,13]. The gradient refractive index of the metallic parallel plate cells can be obtained by gradually changing or modifying their geometrical parameters. The refractive index of the constituent material varies in the range of 1–$\sqrt{2}$ for classical Luneburg lenses and 1–2 for Maxwell fisheye lenses [14]. In [15], a low-loss, thin (λ/4) Luneburg lens based on the Sievenpiper [16] “mushroom” structure was fabricated and characterized at microwave frequencies. Recently, glide-symmetric half-height pins embedded in two mirrored metal plates were used to design a wideband low-profile Luneburg lens [17]. A wideband Luneburg lens based on air-filled glide-symmetric holes and pins was presented in [18]. The effective refractive index was calculated in two propagation directions to illustrate cell anisotropy in [18]. The effective scalar refractive indices (*n_eff_*) of these lenses [15,17,18,19,20,21] were predicted from the dispersion diagram of the quasi-transverse electromagnetic (TEM) mode (eigenmode method). As reported in [6], a 2D broadband low-loss Luneburg lens was designed based on complementary metamaterials. The scalar effective permittivity (*ε_eff_*) and permeability (*μ_eff_*) of this lens were obtained from the TEM transmission line scattering parameters of its unit cell. The dependences of the effective refractive index on the shape, size, and concentration of the conducting elements were investigated for a normal incidence in [22]. Using the same procedures, the effective refractive index of a sub-wavelength periodic-structured unit cell was calculated [23]. However, these investigations were limited to just observing the scalar effective parameters. The eigenmode method relies on calculating the eigenfrequencies for specified values of the phase constant (β) for the quasi-TEM mode [24]. Regardless of the simplicity of the eigenmode method, it only derives the scalar collective effective refractive index and considers *μ_eff_* to be a unity, and *n_eff_* is only related to *ε_eff_* [17,25,26]. This limitation also affects the determination of the actual value of the wave impedance (*Z_eff_*) and, hence, the impedance matching. Similarly, methods based on scalar scattering parameters employed to derive scalar effective parameters neglect the anisotropy characteristics and the directional impedance effects.

In general, metallic and similarly structured lenses are anisotropic materials. Quantities such as the level of anisotropy, the actual value of *μ_eff_*, impedance matching, losses, the directional *n_eff_*, as well as the tensorial permittivity and permeability must be accurately characterized for metallic lenses and their correct operation performance.

This paper presents a full free space anisotropic characterization of metallic lenses. The complex effective EM properties (*n_eff_* and *Z_eff_*) are extracted from the free space reflection and transmission coefficients of the periodic structure. The directional *n_eff_* and *Z_eff_* are used to accurately determine the tensorial permittivity and permeability parameters of the metallic unit cells. It is demonstrated that the magnetic permeability is frequency dispersive in contrast to that shown in previous works based on scalar measurement methods. Essential factors (the level of anisotropy, impedance matching, losses, and incidence angle operation) that affect the design of the lenses were evaluated and compared for different lens structures. A case study of a flat-metallic lens is assessed to demonstrate the effect of these factors on the lens’s performance.

## 2. Free-Space Anisotropy Characterization

**Figure 1:**
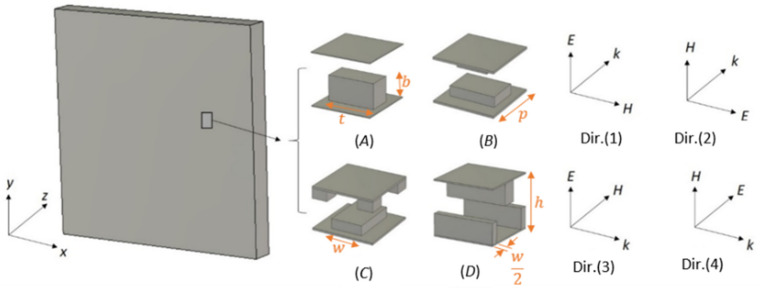
The investigated unit cells with their slab plane perpendicular to the wave incidence at four propagation directions. (*A*) Unit cell *A*, (*B*) unit cell *B*, (*C*) unit cell *C*, and (*D*) unit cell *D*.

Lenses comprising metallic periodic cells are employed to focus EM energy from multiple point sources into a sharp beam. Therefore, characterizing the cells of these lenses as isotropic is inadequate, and there is a crucial need to determine their effective tensorial parameters. In this section, the tensorial EM parameters of four well-studied metallic unit cells were obtained using a full free-space anisotropic characterization (anisotropic characterization method). Figure 1 shows the four-unit cells with plane wave excitation. The unit cell is periodically repeated on the plane that is perpendicular to the incidence direction. The figure describes the directions of the electric field E, magnetic field H, and the propagation constant *k*. The propagation directions are in the z and x directions, and each direction involves both vertical (E is along the y direction) and horizontal (H is along the y direction) polarizations. The unit cell (*A*) has two parallel plates with one pin. The unit cells (*B*) and (*C*) have two and five pins, respectively. The unit cell (*D*) is a glide-symmetric unit cell. All cells have a thickness *p* = 0.715 mm where the thickness of their parallel plates is 18 μm. The cell and pin heights for cell (*A*) are *h* = 0.85 *p* and *b* = 0.5 *h*, respectively. The width (*w*) and thickness (*t*) of the pin in cell (*A*) are 0.47 *h* and 0.9 *h*, respectively. For unit cells (*B*) and (*C*), *h* = 0.5 *p*, *w* = *h*, *t* = 1.5 *h*, and *b* = 0.25 *h*. For unit cell (*D*), *h* = *p*, *w* = 0.25 *h*, and *b* = 0.4 *h*.

**Figure 2:**
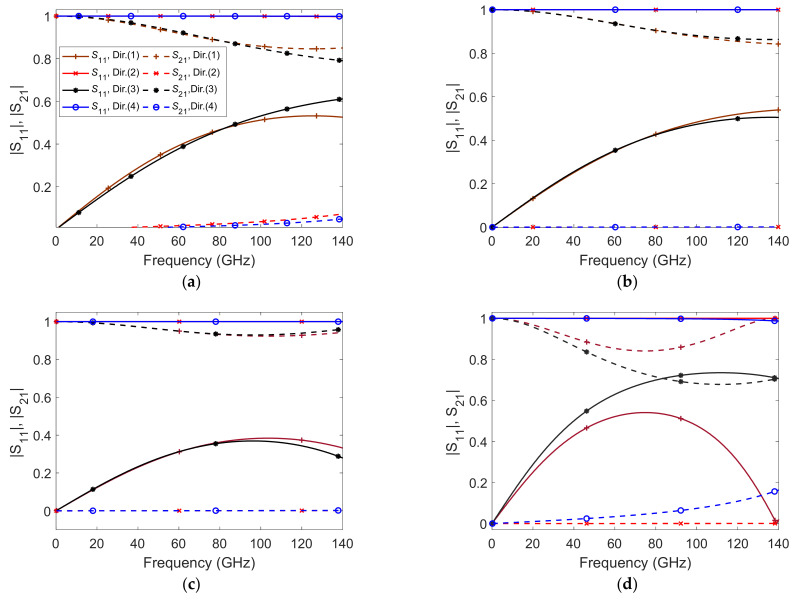
Scattering parameters of the unit-cells *A*, *B*, *C* and *D* at four propagation directions. (**a**) Unit cell *A*, (**b**) unit cell *B*, (**c**) unit cell *C*, and (**d**) unit cell *D*.

The effective parameters *n* and *Z* and the corresponding tensorial $\tilde{\varepsilon }$ and $\tilde{\mu }$ of these metallic cells are determined from the free-space reflection and transmission data at N distinct frequency points. The free-space reflection and transmission data are numerically computed using a full wave electromagnetic simulator. Figure 2 shows the magnitude of the scattering parameters at all depicted polarizations for cells (*A*), (*B*), (*C*) and (*D*).

The magnitudes of the scattering parameters of Figure 2 show that these cell structures interact differently with different polarized EM fields, especially those of cell (*D*). This behavior indicates actual anisotropic characteristics.

Generally, a homogeneous anisotropic material slab possesses constitutive EM parameters that are expressed as follows [27]:

(1) $$D=\tilde{\varepsilon }E\;B=\tilde{\mu }H$$

where $\tilde{\varepsilon }$ and $\tilde{\mu }$ are 3 × 3 elements expressed as:

$$\tilde{\varepsilon }=\left[\begin{array}{c} \begin{array}{ccc} \varepsilon_{x} & -j\varepsilon_{xy} & -j\varepsilon_{xz} \end{array}\\ \begin{array}{ccc} j\varepsilon_{yx} & \varepsilon_{y} & -j\varepsilon_{yz} \end{array}\\ \begin{array}{ccc} j\varepsilon_{zx} & j\varepsilon_{zy} & \varepsilon_{z} \end{array} \end{array}\right],\;\tilde{\mu }=\left[\begin{array}{c} \begin{array}{ccc} \mu_{x} & -j\mu_{xy} & -j\mu_{xz} \end{array}\\ \begin{array}{ccc} j\mu_{yx} & \mu_{y} & -j\mu_{yz} \end{array}\\ \begin{array}{ccc} j\mu_{zx} & j\mu_{zy} & \mu_{z} \end{array} \end{array}\right].$$

For the metallic lenses modeled in this paper, the electromagnetic properties of the unit cells can be described by the local effective medium theory because their thickness is much smaller than the operating wavelength. The nonlocal effects are usually considered for plasmonic structures and at optical frequencies, especially for multi-layered structures, and are negligible in most natural materials or in artificial metallic and dialectic materials with thicknesses much smaller than the operating wavelength [28,29,30]. The retrieval of the tensorial permittivity and permeability commenced with the deduction of the values of *n_eff_* and *Z_eff_* that were obtained from the four directed transmission and reflection measurements. The relationships between the scattering parameters (*S*_11_ and *S*_21_) and *Z_eff_* and *n_eff_* are given by the following relations [31,32,33]:

(2) $$Z_{eff}=\pm \sqrt{\frac{{\left(1+S_{11}\right)}^{2}-S_{21}{}^{2}}{{\left(1-S_{11}\right)}^{2}-S_{21}{}^{2}}}$$

(3) $$n_{eff}=\frac{-i}{k_{0}d}\;ln\left(\frac{S_{11}}{1-S_{21}\left(\frac{Z_{eff}-1}{Z_{eff}+1}\right)}\right),$$

where *d* is the thickness of the material slab and *k*_0_ is the free-space wavenumber.

*ε_eff_* and *μ_eff_* are related to *n_eff_* and *Z_eff_* through the expressions ε*_eff_* = *n_eff_*/*Z_eff_* and *μ_eff_* = *n_eff_ Z_eff_* [34].

For passive metamaterials, the real value of *Z_eff_* and the imaginary value of *n_eff_* must be ≥0 [32]. Therefore, the sign of *Z_eff_* must be determined according to those conditions. From the symmetry of these unit cells and the resultant lossless values of the permeability and permittivity, the tensorial $\tilde{\varepsilon }$ and $\tilde{\mu }$ of these unit cells can be represented practically as biaxial anisotropic tensors, as follows:

(4) $$\tilde{\varepsilon }=\left[\begin{array}{c} \begin{array}{ccc} \varepsilon_{x} & 0 & 0 \end{array}\\ \begin{array}{ccc} 0 & \varepsilon_{y} & 0 \end{array}\\ \begin{array}{ccc} 0 & 0 & \varepsilon_{z} \end{array} \end{array}\right],\;\tilde{\mu }=\left[\begin{array}{c} \begin{array}{ccc} \mu_{x} & 0 & 0 \end{array}\\ \begin{array}{ccc} 0 & \mu_{y} & 0 \end{array}\\ \begin{array}{ccc} 0 & 0 & \mu_{z} \end{array} \end{array}\right]$$

The above formulation is used in a full free space anisotropic characterization of the selected metallic cell structures.

**Figure 3:**
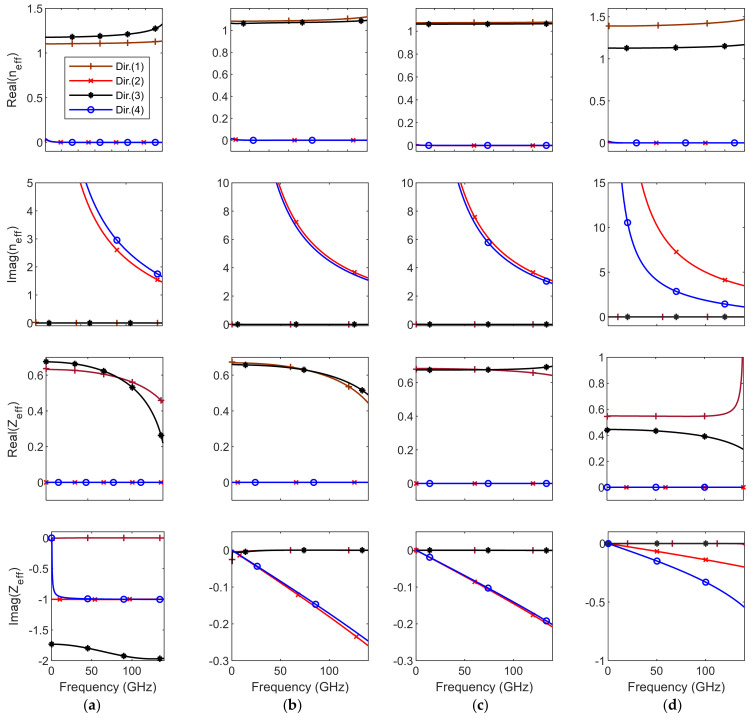
The extracted *n_eff_* and Z*_eff_* parameters of unit cells *A*, *B*, *C* and *D* at four propagation directions. (**a**) Unit cell *A*, (**b**) unit cell *B*, (**c**) unit cell *C*, and (**d**) unit cell *D*.

Figure 3 shows the corresponding directional *n_eff_* and *Z_eff_* of the four investigated unit-cells. Clearly, *n_eff_* and *Z_eff_* are pure real for vertical polarization and pure imaginary for horizontal polarization. *n_eff_* and *Z_eff_* for unit cells (*C*) and (*D*) show less dispersive behavior than other unit cells. The anisotropy level of these unit cells is different and depends on the cell directional structure. The GRIN materials are typically designed to aim at nonresonant low frequencies because they are less lossy, nondispersive, and practical at such frequencies, especially at a normal incidence propagation.

However, the antenna radiation rays towards the lens are usually non-normal except in the small region at the center of the lens. Hence, it is important to determine the anisotropy effects in the lens characterization process. More anisotropy is manifested in the lenses for multiple beam antennas.

**Figure 4:**
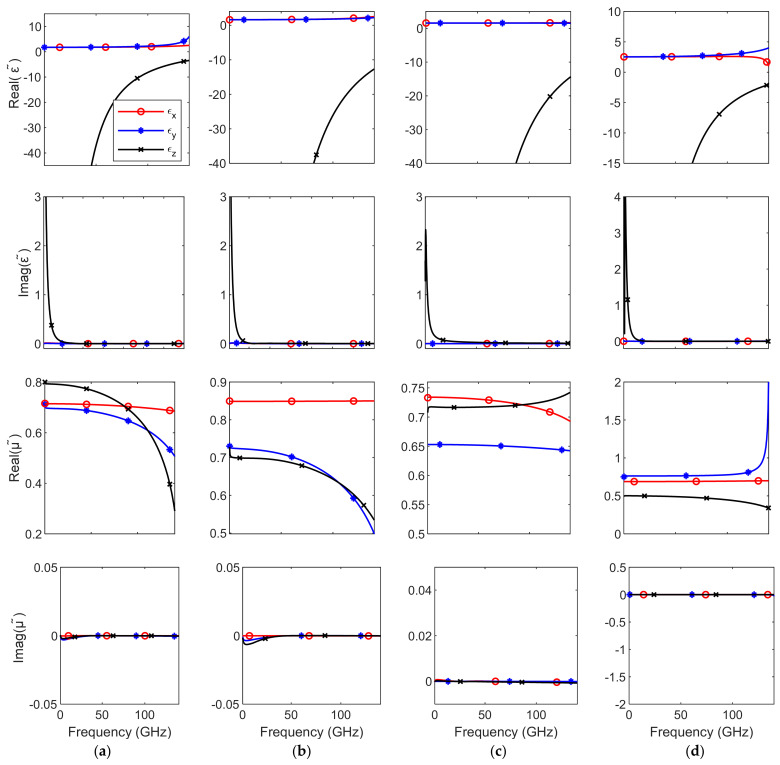
The extracted tensorial $\tilde{\varepsilon }$ and $\tilde{\mu }$ of the unit cells *A*, *B*, *C* and *D*. (**a**) Unit cell *A*, (**b**) unit cell *B*, (**c**) unit cell *C*, and (**d**) unit cell *D*.

The anisotropic tensorial permittivity and permeability have been calculated from the directional values of the refractive index and wave impedance. The directional refractive index and wave impedance have been calculated from the reflection and transmission coefficients at different directions and polarizations. The results in Figure 4 indicate that the behaviors of the permittivity and permeability are biaxial anisotropic with different levels of anisotropy in the four cells. The tensor elements *ε_x_*, *ε_y_*, *ε_z_*, *μ_x_*, *μ_y_*, and *μ_z_* are real and frequency-dispersive. The results obtained based on the classical eigenmode method yield single culminated scalar quantities and do not show any directional tensorial behavior. The magnetic permeability *μ_eff_* is usually ignored and set to one in such methods; hence, both ε*_eff_* and *Z_eff_* are calculated loosely from *n_eff_* [17,25,26].

## 3. Result Comparisons

In this section, the studied unit cells shown in Figure 1 were characterized using the anisotropic characterization method and the eigenmode method. To realize the comparison of the results, only the effective parameters *n_eff_*, *Z_eff_*, *ε_eff_*, and *μ_eff_* were computed from the proposed free space characterization, since the other method does not describe anisotropy and does not provide $\tilde{\varepsilon }$ and $\tilde{\mu }$ tensors. The extracted effective EM parameters are computed for a changing cell geometry at 60 GHz. The lens is designed for a vertical polarization operation. For the unit-cells, the main parameter that changes the properties of their structure is the ratio of the height of the metallic pin to the distance between parallel plates (*b*/*h*).

The eigenmode method considers the effective μ to be a unity, and the effective wave impedance and effective permittivity are calculated directly from the effective refractive index of the quasi-TEM mode.

**Figure 5:**
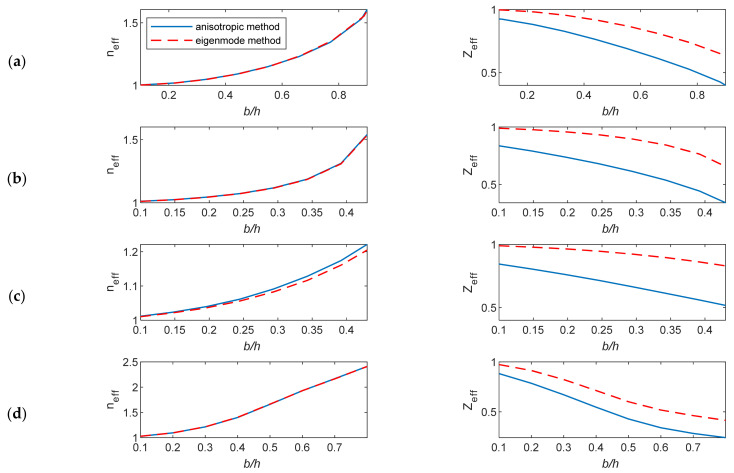
The extracted *n_eff_* and *Z_eff_* versus the ratio *b/h* for the unit cells *A*, *B*, *C* and *D*. (**a**) Unit cell *A*, (**b**) unit cell *B*, (**c**) unit cell *C*, and (**d**) unit cell *D*.

**Figure 6:**
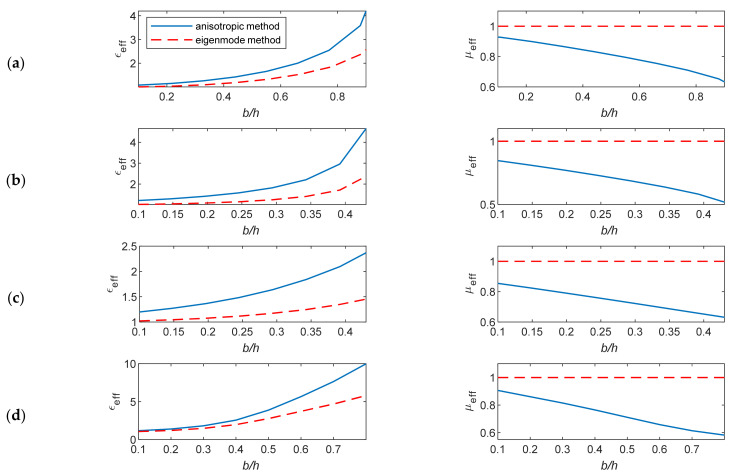
The extracted ε*_eff_* and *μ_eff_* versus the ratio *b/h* for the unit cells *A*, *B*, *C* and *D*. (**a**) Unit cell *A*, (**b**) unit cell *B*, (**c**) unit cell *C*, and (**d**) unit cell *D*.

This consideration affects the determination of the actual value of the impedance matching at the lens-to-air boundary. The anisotropic characterization method is based on the inverse calculations of *n_eff_* and *Z_eff_* from the transmission and reflection coefficients. The corresponding ε*_eff_* and *μ_eff_* were calculated from the obtained *n_eff_* and *Z_eff_*, and these calculations were repeated for different pin heights. The EM parameters were plotted against *b*/*h* at 60 GHz, as shown in Figure 5 and Figure 6.

The extracted wave impedance based on the anisotropic characterization method is always less than that calculated based on the eigenmode method. This difference is more evident as *b*/*h* increases. The results show different levels of mismatch at the lens–air boundary in the studied cell structures as the value of *Z_eff_* is <1. Such accurate *Z_eff_* values are not obtainable using the eigenmode method. The height of unit cells and the thickness of the parallel plates affect the impedance matching. The impedance information is obtained correctly using the anisotropic characterization method, while the eigenmode method gives constant impedance values for different plate thicknesses. 

The anisotropic characterization method provides a true frequency-dispersive effective permittivity and permeability.

The differences in the effective permittivity based on these two methods can increase when the ratio *b/h* increases due to the decreasing values of the effective permeability *μ_eff_*, which is ignored in the eigenmode method.

**Figure 7:**
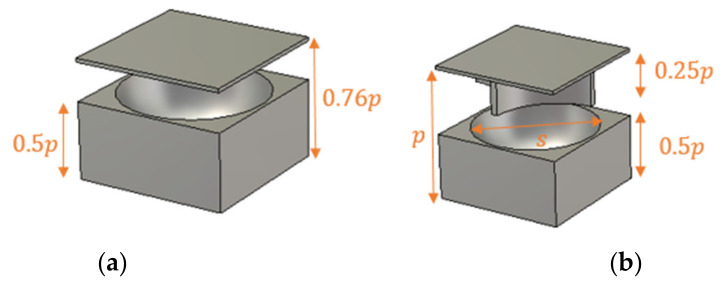
(**a**) Holey and (**b**) holey glide-symmetric unit cells.

The dissimilarity in the retrieved parameters from the characterization methods is more significant in more complicated structure unit cells (Figure 7).

**Figure 8:**
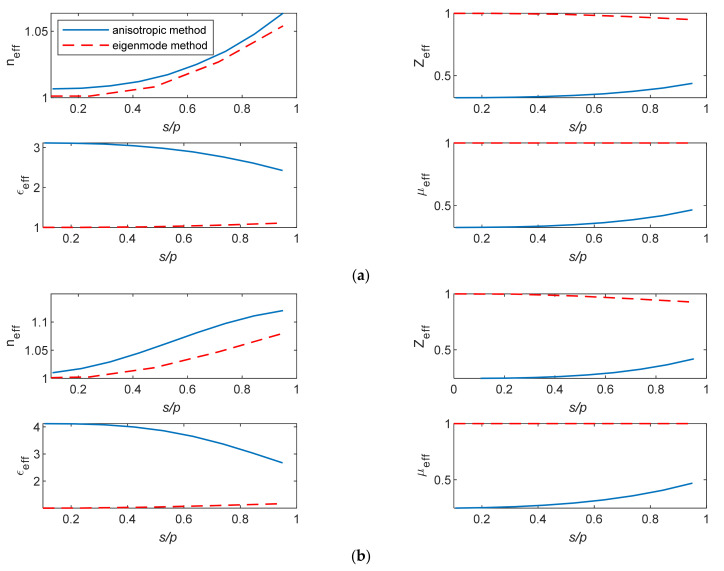
The extracted EM parameters of (**a**) holy and (**b**) holy glide-symmetric unit cells.

Figure 8a,b shows the EM parameters versus the diameter (*s*) of the air hole of the cells in Figure 7 at 60 GHz. The eigenmode method leads to inaccurate information about the EM behavior of such cells and will result in erroneous lens intricate designs.

## 4. Anisotropy Effects on Lens Design

In this section, the effect of anisotropy on the effective EM parameters and losses in the unit cells (*A*), (*B*), (*C*), and (*D*) of Figure 1 are studied at different incidence angles. The incidence angles from the source antenna beam to the lenses are not normal except at the center of the lens, especially in the case of multi-beam antenna sources. The incidence angle is oblique in one plane (*ϕ*) in circular metasurface lenses employed to design a waveguide crossover and multi-beam antennas [35,36] and oblique in two planes (*ϕ* and *θ*) in flat lenses [37,38]. The *ϕ* and *θ* represent the azimuthal angle and polar angle of the standard spherical coordinate system of the lens structure shown in Figure 1.

**Figure 9:**
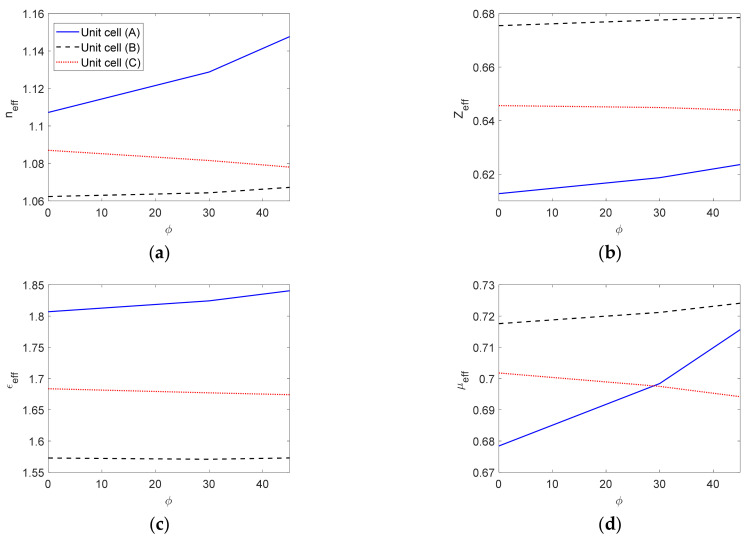
Effective EM parameters versus *ϕ* for the unit-cells *A*, *B*, and *C*. (**a**) The effective refractive index, (**b**) the effective wave impedance, (**c**) the effective permittivity, and (**d**) the effective permeability.

The unit-cells (*A*), (*B*), and (*C*) can be employed to design a circular lens at 60 GHz. The incidence angle *ϕ* varies in the range of 0–45°. Figure 9 illustrates how the corresponding effective EM parameters change when modifying *ϕ*. The main contributor to changing the EM parameters with *ϕ* is the metallic pins inserted between the parallel plates. The level of anisotropy in the different structures plays a prime role in the parameter variations with the angle of incidence. Cell (*A*) exhibits more evident parameter value changes with *ϕ* than the other cells. Additionally, changes in the effective refractive index and effective permeability are more evident than the effective wave impedance and effective permittivity.

**Figure 10:**
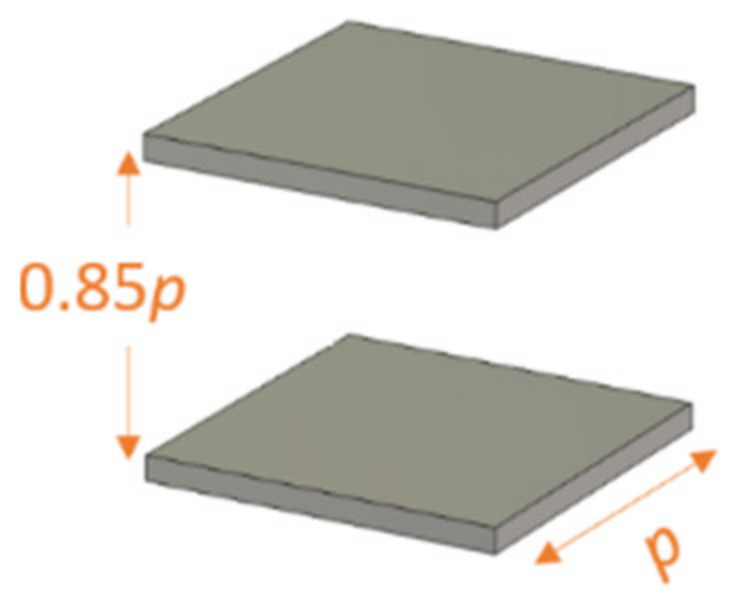
Two empty parallel plates unit cell (*E*).

For a flat lens, unit-cells (*A*), (*B*), (*C*), and (*D*) can be employed [36]. Here, besides the cells (*A*), (*B*), (*C*), and (*D*), a unit cell consisting of two empty parallel plates (unit cell *E*), as shown in Figure 10, is used to investigate the effect of anisotropy and losses at different incidence angles *θ*.

**Figure 11:**
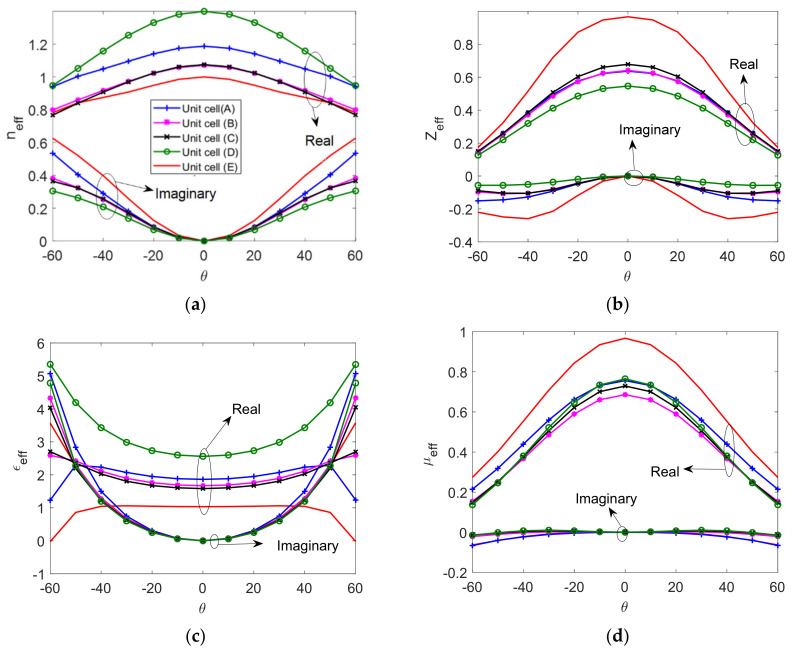
Effective EM parameters versus *θ*. (**a**) The effective refractive index, (**b**) the effective wave impedance, (**c**) the effective permittivity, and (**d**) the effective permeability.

Figure 11 shows the effective EM parameters of the five investigated unit-cells (*A*), (*B*), (*C*), (*D*) and (*E*) at different incident angles *θ*. Both the real and imaginary parts of the effective EM parameters respond variously with *θ* variations to indicate different levels of anisotropy. The imaginary part of the effective EM parameters indicates the loss introduced in the oblique incident propagation. The magnetic permeability varies with *θ* and is less than unity. In addition, the impedance matching obviously changes when increasing *θ*.

In the case of a vertical polarized wave incidence—as studied here—the cell structures with transverse metallic plates and pins are less sensitive to variations in the incidence angle *ϕ* than the incidence angle *θ*, which explains the apparent change in the effective EM parameters.

At a normal incidence, the simple empty parallel plate unit cell has similar characteristics to those derived using the eigenmode method. At an oblique incidence, *n_eff_* and the other EM parameters change with the incidence angle *θ*. This shows that the anisotropy that results from the parallel plates is significant. This is not observed using the eigenmode method. These effects are even more apparent in more complex unit cells, as discussed in the results shown above. Therefore, the design of metallic lenses based on just a simple eigenmode characterization is not adequate and may lead to incorrect results. The following case study explains the effect of anisotropy and the other mentioned factors on the performance of the lens.

## 5. Flat Lens Example

**Figure 12:**
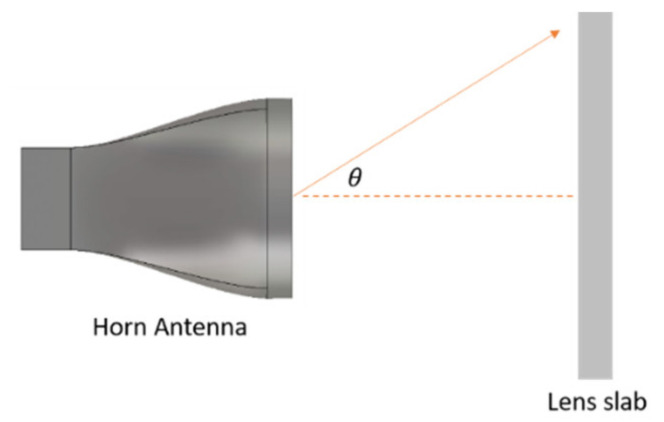
Horn antenna with a flat lens.

**Figure 13:**
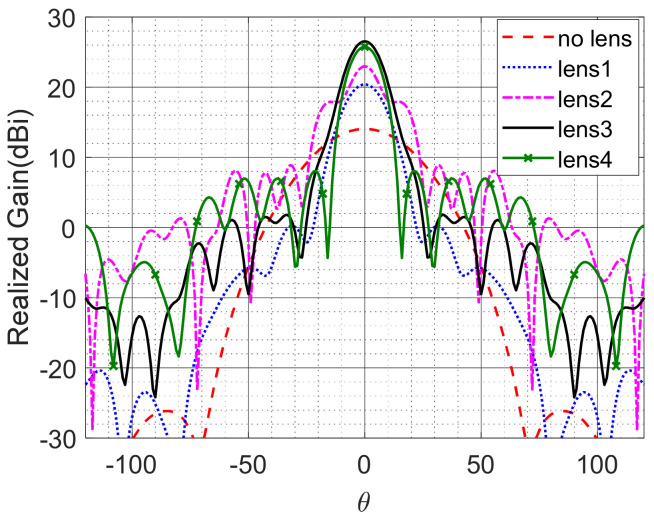
Radiation pattern of the horn antenna without and with flat lenses.

Figure 12 shows a horn antenna with a flat lens. In this section, we illustrate that the simple eigenmode characterization of unit cells is inadequate and that the anisotropy characterization is crucial. Figure 13 shows the radiation pattern of the horn antenna at 60 GHz with and without different flat lenses.

Four different lenses were studied in this example. The first one represents a dielectric Luneburg lens that consists of six concentric rings, each with a different *ε* value. This design is used as a reference for comparison. The second lens was constructed using the empty parallel plate cell. The eigenmode method characterizes this type by *n_eff_* with a unit value, which predicts that such a lens would have no effect or contribution to the antenna radiation. Figure 13 illustrates that this lens both contributed about 9 dB to the focus of the main beam of the horn antenna and altered the side lobe level. This is mainly attributed to the anisotropy effects that cause grading in the lens’s effective refractive index.

The third lens is a hybrid dielectric Luneburg lens inserted in an empty parallel plate cell. The expected results based on the eigenmode method should be similar to those of the reference lens (lens 1). However, the anisotropy of the parallel plates clearly enhances the realized gain. The fourth lens is built using a unit cell (*D*) filled with a dielectric Luneburg lens. The eigenmode method predicts a uniform spatial increment in the refractive index at all points on the flat lens due to the metallic cells, and so the expected results should be similar to those of the reference lens. However, the results are different, and the effect of the anisotropy of the unit cell (*D*) deteriorates the side lobe level by about 7 dB. The effects of the loss and the impedance mismatching obviously appear when increasing the side lobe levels of lenses 2, 3 and 4 as compared to a lossless dielectric lens (lens 1). Considering the correct characterization of the effect of anisotropy, losses, impedance matching, and the actual value of the magnetic permeability yields a more accurate lens design and optimization in different applications.

## 6. Conclusions

This paper explores the full electromagnetic characterization of metallic lenses. The tensorial EM parameters of metallic lenses were acquired from the free-space transmission and reflection coefficients. The permittivity and permeability tensors for four different metallic cells are derived from the effective refractive and the wave impedance directional vectors. The investigated cells show different levels of anisotropy and a less than unity frequency-dispersive magnetic permeability for the four cells. The wave impedance is highly dispersive, which will affect the lens-to-air impedance matching. Such characteristics of metallic lenses could not be predicted by the eigenmode method typically used in lens applications. The full anisotropy characterization of these cells and of complex holy and holy glide-symmetric cells yields accurate effective refractive index, wave impedance, permittivity, and permeability values, unlike those obtained from a simple eigenmode method or simple normal incidence characterization. The effect of variations in the incidence angles is manifested by remarkable modifications for all effective parameter values, particularly demonstrated as anisotropy, losses, impedance matching, and dispersive magnetic permeability effects. In the flat lens case study, the effect of the anisotropy due to angle variations in the incident wave on the refractive index, impedance matching, losses and hence on the performance of the metallic lens design and operation was verified.

## Data Availability

Not applicable.
